# Optimal design of impeller for self-priming pump based on orthogonal method

**DOI:** 10.1038/s41598-023-43663-0

**Published:** 2023-10-01

**Authors:** Yu-Liang Zhang, Kai-Yuan Zhang, Zu-Chao Zhu

**Affiliations:** 1 College of Mechanical Engineering and Key Laboratory of Air-Driven Equipment Technology of Zhejiang Province, Quzhou University, Quzhou, 324000 China; 2 School of Mechanical Engineering, Hunan University of Technology, Zhuzhou, 412007 China; 3 The Zhejiang Provincial Key Lab of Fluid Transmission Technology, Zhejiang Sci-Tech University, Hangzhou, 310018 China

**Keywords:** Energy science and technology, Engineering

## Abstract

In order to improve the efficiency of the self-priming pump in the outdoor emergency rescue mobile pump truck, this paper took the key energy conversion component-impeller as the target and used the orthogonal experimental design method to optimize its hydraulic performance. Firstly the numerical calculations were compared with the experimental results to confirm the reliability of the calculation method. Then, *L*_9_ (3^4^) orthogonal design was applied to investigate the influence of the impeller diameter, the blade outlet width, the blade wrap angle and the number of blades on the hydraulic performance of the self-priming pump. Through range analysis, the order of influence of each influencing factor on the head and efficiency of the self-priming pump was determined, and finally obtained the optimal parameter combination scheme. The results show that the optimized self-priming pump exceeds the head of the prototype pump at all flow conditions, and the efficiency curve at high flow conditions is significantly improved and has a wide high efficiency zone.

## Introduction

As a special kind of centrifugal pump, the self-priming pump contains obvious shape features such as gas–liquid separation chamber and return hole. In comparison with ordinary centrifugal pumps, the self-priming function of self-priming pumps makes them widely used in flooding, irrigation and chemical industry. In recent years, many scholars at home and abroad have conducted in-depth research on the relationship between the geometric parameters of self-priming pumps and their performance. Luo et al.^1^ combined orthogonal experimental methods and gray correlation method to investigate the influence of structural parameters such as blade thickness, impeller inlet and outlet angles, the wrap angle and splitter blade diameter on the hydraulic performance of the centrifugal pump. Through data analysis, the optimal combination of parameters for the corresponding evaluation indexes was derived. Chang et al.^2,3^ established a new model for predicting self-priming performance, and analyzed the influence of nozzle geometric parameters on the performance of jet self-priming pump using orthogonal experimental method and grey correlation method. They also achieved to improve the self-priming performance by changing the nozzle geometry, and finally tested the pump with optimal parameters at different self-priming heights to verify its reliability. Zhang et al.^4^ proposed a multiobjective optimization design based on the combination of numerical simulation technology and orthogonal experiments. The number of blades, the blade setting angle, the hub ratio, and the distance between the blade and the guide vane were identified as influencing factors in the orthogonal test scheme. The head, efficiency, shaft power and pressure pulsation were identified as quality indicators. The simulation results showed that the final optimized solution had significant improvements in all quality indicators compared to the prototype pump. Based on the orthogonal design principle, Wang et al.^5^ explored the influence of blade groove structure on centrifugal pump performance by replacing the experimental process with numerical simulation calculations. The results showed that the slot width and slot depth are the main influencing factors on the hydraulic performance of the pump under low flow conditions. And the effect of slotting position on hydraulic performance under high flow conditions is obvious. Li et al.^6^ also took advantage of numerical simulation techniques to replace the real experimental process. Based on the principle of orthogonal design, the blade outlet width, the blade outlet angle and the blade wrap angle were selected as influencing factors to optimize the hydraulic performance of high specific speed centrifugal pumps, while also taking into account the anti-cavitation performance of the centrifugal pump. The numerical simulation results showed that under high flow conditions, the optimized centrifugal pump performance has a more significant improvement. Hou et al.^7^ proposed a performance optimization scheme for centrifugal pumps based on local entropy generation theory and combined with orthogonal experimental method. They selected blade outlet setting angle, the wrap angle, volute inlet width, and throat area as the influencing factors to establish the orthogonal scheme. After determining the optimal combination of parameters, the numerical results showed a slight increase in pump efficiency and a significant reduction in entropy production. With the combination of experiments and numerical simulations, Ding et al.^8^ explored the effect of variation of blade outlet angle on the internal flow field and hydraulic performance of a high specific speed centrifugal pump. According to the comprehensive analysis, the blade outlet angle had more significant effect on pump efficiency and less effect on pump head. Under high flow conditions, increasing the blade outlet angle significantly increased the hydraulic loss of the impeller, resulting in lower efficiency. Yousefi et al.^9^ addressed the issue of significant performance degradation of centrifugal pumps when transporting oil media. The effect of geometric features such as impeller inlet and outlet angles and the blade edge shape on its performance was investigated by numerical simulation. The simulation results showed that it operates better when the blade outlet angle increased in a certain range. When conveying thick oil media, an oval shape at the blade inlet with an angle of 45° and a rounded outlet with an angle of 40° was the best combination of parameters for performance. Ayremlouzadeh et al.^10^ used Taguchi optimization method to improve the performance of a low specific speed centrifugal pump. The results showed that the blade outlet width and blade outlet angle were important factors in enhancing shaft power and efficiency, especially the blade outlet width. And the parameter combinations with minimum power and maximum efficiency were determined by combining with ANOVA, respectively. Elyamin et al.^11^ focused on the effect of the blade number on the hydraulic performance of centrifugal pumps by means of numerical calculations. The results showed that the low blade number would lead to poorer flow stability and increased mixing losses at the impeller outlet. In contrast, too many blades led to a weaker tendency of energy stratification, which was more likely to generate wake and increased friction losses. By the combination of experiments and numerical simulations, Namazizadeh et al.^12^ optimized the overall performance of centrifugal pump by adding different geometries of splitter blades to the impeller. Then they employed the Design of Experiment (DOE) technique and response surface methodology to obtain the optimal geometry, which led to improvements in head and efficiency. Gao et al.^13^ used a four-factor three-level orthogonal experimental design method to optimize the geometric parameters of an open vortex pump, and verified the optimization by experiments. After analyzing the data from the experiments and numerical simulations, the primary and secondary factors affecting the hydraulic performance of the vortex pump were identified. The efficiency and head of the optimized model were improved compared to the prototype pump. In addition, it was found that due to the open impeller structure of the vortex pump, the length of the rotating recirculation generated in the impeller would gradually decrease as the flow rate increased. Zhao et al.^14^ conducted a multi-objective orthogonal optimization design of a tubular pump while maintaining low shaft power. After using a combination of range analysis and weighted matrix method, the optimal combination of geometric parameters such as the number of blades, airfoil type, blade thickness and guide blade spacing was found. The optimized tubular pump had a wide high-efficiency range. Pei et al.^15^ improved the cavitation performance of centrifugal pumps based on an orthogonal test method. By combining the results of numerical simulations and experiments, it was concluded that the impeller inlet diameter had the greatest effect on the cavitation performance of the pump. After optimization, the cavitation performance was significantly improved with a smaller decrease in pump efficiency and the fluid could enter the impeller more smoothly. In summary, the impeller is the most critical over-flow component in the pump, and the determination of its structural parameters directly affects the performance of the pump. By using the orthogonal design method, scholars at home and abroad can scientifically and effectively improve the performance of centrifugal pumps by changing the geometric parameters and determining the best parameter combination scheme. However, there are fewer studies on the parameter optimization of self-priming pump impeller. Self-priming pumps have special structures such as gas–liquid separation chamber and reflux hole, which are obviously different from ordinary centrifugal pumps. The optimization object of this article is a horizontal self-priming reinforced pump installed on a mobile pump truck for emergency rescue. It is of great significance to improve efficiency and save energy in outdoor emergency rescue. This article is based on the principle of orthogonal design to determine the critical geometric factors and their influencing levels. Numerical calculation method is used to replace the experimental process for research, and the optimal parameter combination scheme is ultimately determined. In the research process, this paper considers the influence of the reflux hole, the front and rear pump chambers and the clearance of wear-ring on the hydraulic performance of the self-priming pump, which are rarely considered in the research process of scholars^6–9^. In addition, the prototype pump belongs to the category of low specific speed, and fewer studies have been carried out on parameter optimization based on the orthogonal design method for the impeller of low specific speed pumps. The research in this paper provides a reference for the hydraulic performance improvement of self-priming pumps.

## Numerical calculation method

### Computational models and mesh

The optimization object of this paper is a DKS18-40-3 split self-priming reinforced pump, which belongs to the category of external mixed self-priming pump, and its main design parameters are shown in Table 1.

**Table 1:** Table of design parameters of the self-priming pump.

Design parameters	Symbol	Numerical value
Rotational speed	*n*/(r/min)	2900
Flow	*Q*_e_/(m^3^/h)	15
Head	*H*_e_/(m)	32
Power	*P*_e_/(kW)	3
Specific speed	*n*_s_	51

**Figure 1:**
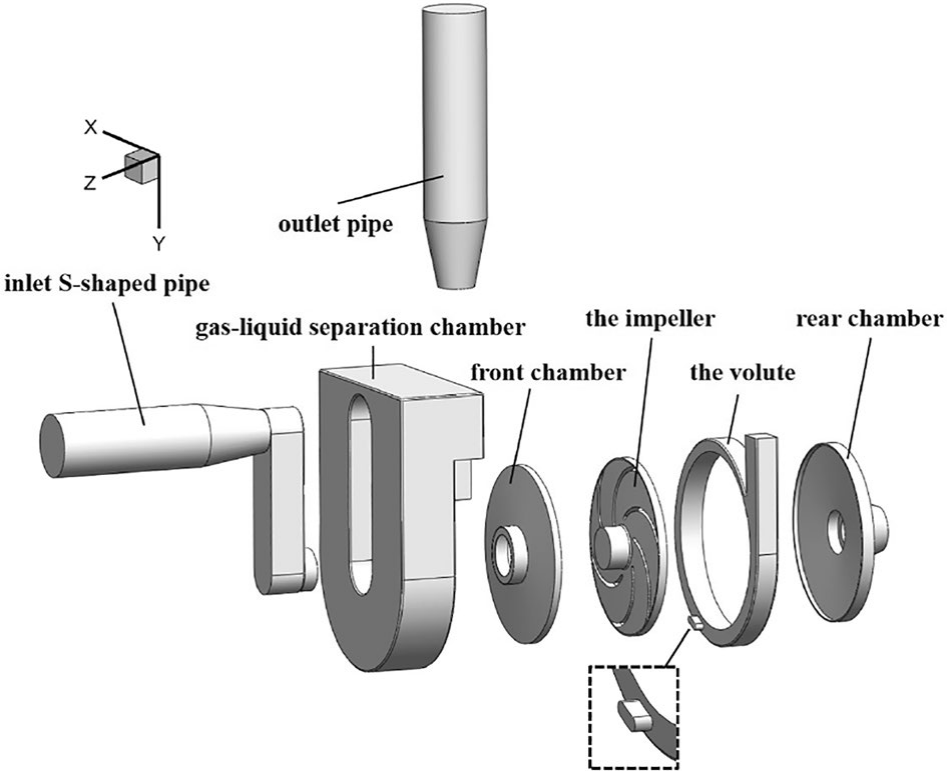
Composition of the calculation domain of the self-priming pump.

The internal flow field calculation domain model of the self-priming pump is shown in Fig. 1. All of the calculated waters include the impeller, the volute, pump front and rear chamber, the inlet S-shaped pipe, gas–liquid separation chamber and the outlet pipe. The water body of the return hole connects the water body of the volute with the lower part of the gas–liquid separation chamber, which is the water storage chamber of the pump. The return hole is located at the position from the volute tongue along the direction of the impeller rotation, which is from 203.81° to 215.88°.

**Figure 2:**
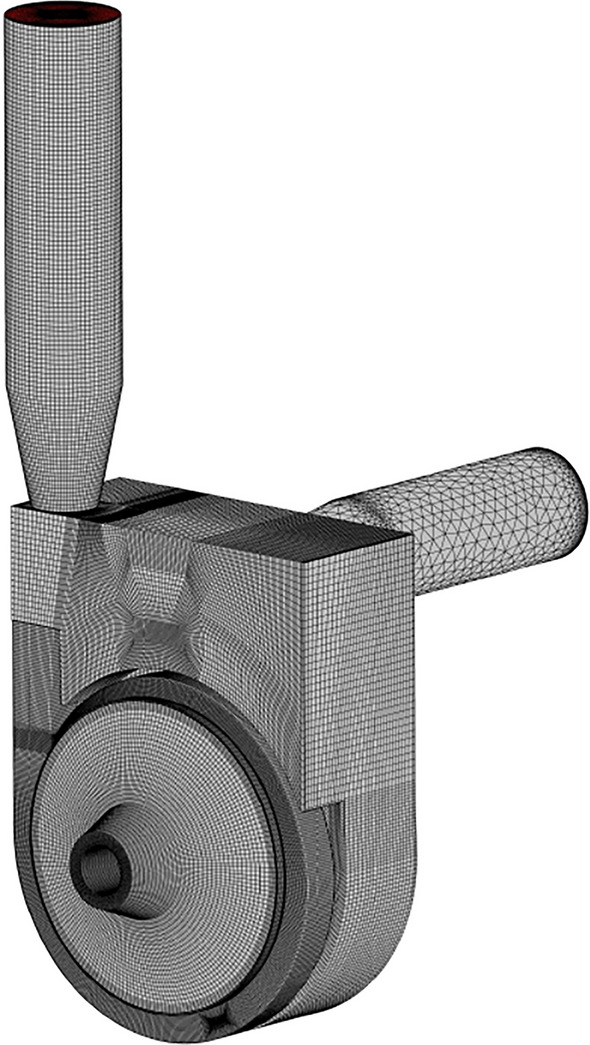
Computational domain mesh.

**Figure 3:**
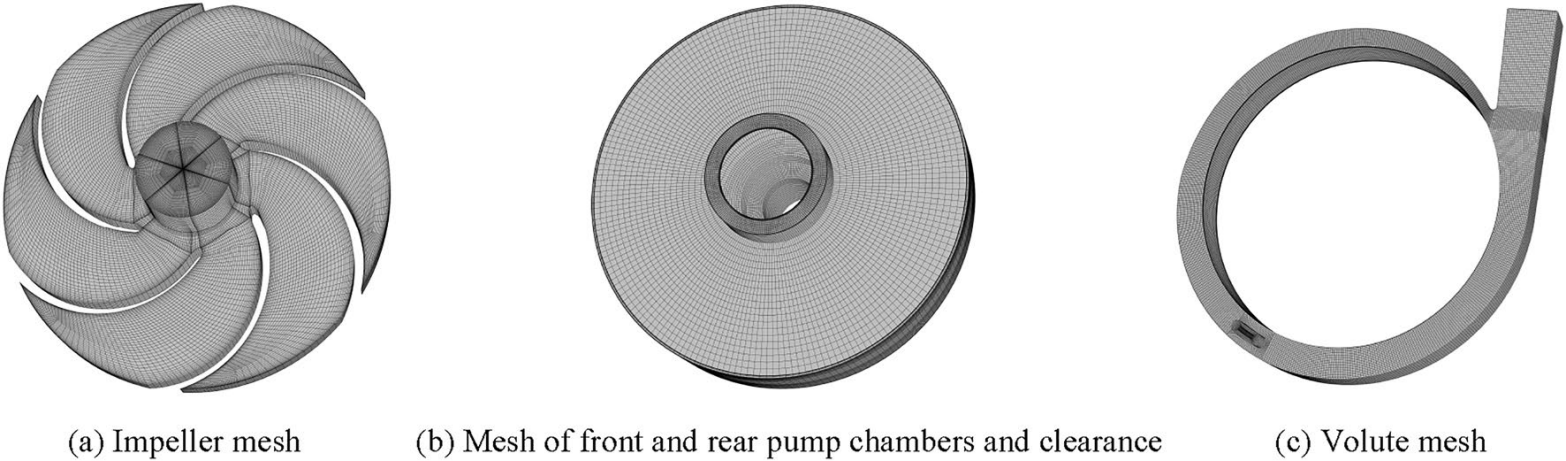
Partial mesh of flow components.

The computational domain mesh was generated by ANSYS19.2-ICEM. In the meshing process, local mesh refining was applied to regions where the flow field changes drastically. To facilitate meshing and reduce the errors that exist in meshing, the front and rear pump chambers, the gap between the impeller and the volute were taken as a whole and then structured meshing was performed. In addition, due to the small geometric features of the inlet S-shaped pipe, unstructured meshing was used to ensure a high mesh quality. The full domain mesh of the self-priming pump is shown in Fig. 2. The structured meshes of the impeller, front and rear pump chambers, and the volute are shown in (a), (b), and (c) of Fig. 3, respectively. The number of meshes, orthogonal quality, and minimum angle for each domain are shown in Table 2.

**Table 2:** Table of mesh information.

Domain name	Mesh number	Orthogonal quality	Minimum angle
S-shaped pipe	213248	0.36	18°
Impeller	396002	0.30	18°
Pump chambers and clearance	292368	0.90	70°
Volute	499744	0.33	27°
Gas–liquid separation chamber	399159	0.29	18°
Outlet pipe	200652	0.75	45°

**Figure 4:**
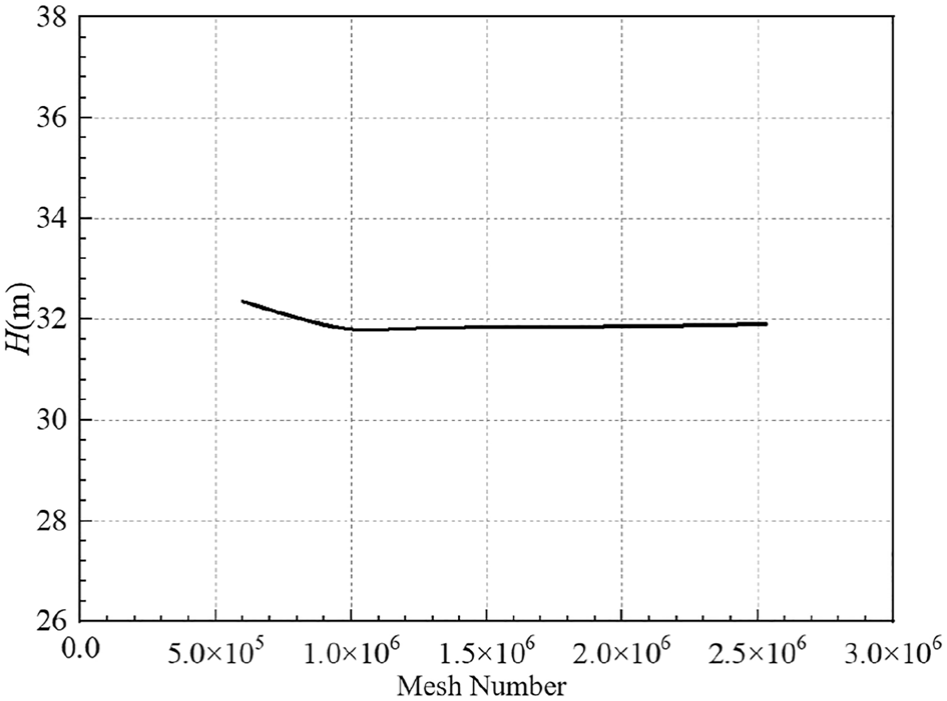
The independence verification of mesh.

The variation of the self-priming pump head with the mesh number under the design flow condition is shown in Fig. 4. It can be seen that as the number of meshes increases, the calculated head tends to decrease and stabilise. When the mesh number increases to about 1.5 million, the numerical head values are basically stable with very small fluctuations. In order to ensure the accuracy of the numerical calculation results, the impeller, the volute and other important flow components with more complex flow fields are encrypted, and the total mesh number is finally determined to be 2001173.

### Control equations

Zhu et al.^16,17^ proved that the Realizable *k–ε* turbulence model is very suitable for pump studies, and the numerical simulation results are in good agreement with the experimental results. Therefore, the numerical simulations in this article adopt the Reynolds-averaged Navier–Stokes (RANS) model, with the Realizable *k–ε* two-equation model used to close the Navier–Stokes equations. The constraint equations for the turbulent kinetic energy *k* and the turbulent dissipation rate *ε* in the Realizable *k–ε* turbulence model are as follows:

1 $$ \frac{\partial (\rho k)}{{\partial t}} + \frac{{\partial \left( {\rho ku_{j} } \right)}}{{\partial x_{j} }} = \frac{\partial }{{\partial x_{j} }}\left[ {\left( {\mu + \frac{{\mu_{{\text{t}}} }}{{\sigma_{k} }}} \right)\frac{\partial k}{{\partial x_{j} }}} \right] + \rho \left( {P_{k} - \varepsilon } \right) $$

2 $$ \frac{\partial (\rho \varepsilon )}{{\partial t}} + \frac{{\partial \left( {\rho \varepsilon u_{j} } \right)}}{{\partial x_{j} }} = \frac{\partial }{{\partial x_{j} }}\left[ {\left( {\mu + \frac{{\mu_{{\text{t}}} }}{{\sigma_{\varepsilon } }}} \right)\frac{\partial \varepsilon }{{\partial x_{j} }}} \right] + \rho C_{1} E\varepsilon - \rho C_{2} \frac{{\varepsilon^{2} }}{{k + \sqrt {v\varepsilon } }} $$

where the coefficient $C_{1} = \max \left( {0.43,\frac{\eta }{\eta + 5}} \right)$, coefficient of eddy viscosity $C_{\mu } = \frac{1}{{A_{0} + A_{s} U^{*} k/\varepsilon }}$. In the equation for the turbulent turbulent eddy viscosity coefficient, $A_{s} = \sqrt 6 \cos \phi$, $\phi = \frac{1}{3}\cos^{ - 1} (\sqrt 6 W)$, $W = \frac{{E_{ij} E_{jk} E_{ki} }}{{\left( {E_{ij} E_{ij} } \right)^{\frac{1}{2}} }}$, $U^{*} = \sqrt {E_{ij} E_{ij} + \tilde{\Omega }_{ij} \tilde{\Omega }_{ij} }$, $\tilde{\Omega }_{ij} = \Omega_{ij} - 2\varepsilon_{ijk} \omega_{k}$, $\Omega_{ij} = \overline{\Omega }_{ij} - \varepsilon_{ijk} \omega_{k}$. The constant values in the above equation are: *σ*_*ε*_ = 1.2, *C*_2_ = 1.92, *A*_0_ = 4.0. The time derivative is zero when calculated as a constant.

### Numerical solution

In this paper, FLUENT 19.2 is used to perform the constant numerical calculations for the hydraulic performance of the self-priming pump. The coupling of the pressure and velocity fields is solved by the SIMPLE method. In the iterative calculation process, the convergence accuracy of velocity components in each direction, turbulent kinetic energy *k*, and turbulent dissipation rate *ε* are all set to the default value of 10^–4^, and the residuals are considered to be converged if they are less than this standard. If the residual calculated during iterations cannot reach the standard, the convergence of the simulation can also be judged by monitoring the stability of performance parameters. In this study, the monitored quantities are the head and impeller torque of the self-priming pump. Data is transferred between different domains through mutual coupling of their interfaces. Among them, the movement of the impeller domain is realized by using the Multiple Reference Frame (MRF) rotation model, while the other water domains remains in a stationary state. The default no-slip boundary condition is used at the solid wall, and the standard wall function is used to treat the near-wall surface.

The velocity inlet boundary is applied to the inlet of the self-priming pump, according to the conservation of mass and the incompressibility of the fluid, the inlet velocity is determined by the flow rate and the inlet diameter, as shown in Eq. (3), while assuming that the tangential velocity and radial velocity of 0. The initial values of *k* and *ε* at the inlet can be determined by the Eq. (4):

3 $$ {\text{v = }}\frac{Q}{A} = \frac{4Q}{{\pi d^{2} }} $$

4 $$ \left\{ \begin{gathered} k = \frac{3}{2}(I\overline{u} )^{2} \hfill \\ \varepsilon = C_{\mu }^{{{3 \mathord{\left/ {\vphantom {3 4}} \right. \kern-0pt} 4}}} \frac{{k^{{{3 \mathord{\left/ {\vphantom {3 2}} \right. \kern-0pt} 2}}} }}{l} \hfill \\ \end{gathered} \right. $$

where, *l* = 0.07*L*, *L* is the characteristic length, which in this paper takes the value of the diameter at the inlet. *I* is the turbulence intensity, $I \approx 0.16{\text{Re}}^{ - 1/8}$, ${\text{Re}} = \frac{{{\overline{\text{u}}} \cdot L}}{{\text{v}}}$, and ${\overline{\text{u}}}$ is the mean velocity at the inlet. $C_{\mu } \approx 0.09$ is the empirical constant in the turbulence model.

In this paper, the free outflow boundary condition is chosen, assuming that the flow is fully developed at the outlet of the computational domain. The velocity components $${\text{u}}_{{{\text{out}}}}$$, pressure $${\text{p}}_{{{\text{out}}}}$$, turbulent kinetic energy $${\text{k}}_{{{\text{out}}}}$$ and turbulent dissipation rate $$\varepsilon_{{{\text{out}}}}$$ are taken as the second type of boundary conditions, i.e. $$\frac{{\left. {\partial u_{j} } \right|_{{\text{out }}} }}{{\partial \vec{n}}} = 0$$, $$\frac{{\partial p_{{\text{out }}} }}{{\partial \vec{n}}} = 0$$, $$\frac{{\partial k_{{\text{out }}} }}{{\partial \vec{n}}} = 0$$, $$\frac{{\partial \varepsilon_{{\text{out }}} }}{{\partial \vec{n}}} = 0$$$$(j = 1,2,3)$$.

### Reliability verification of numerical methods

**Figure 5:**
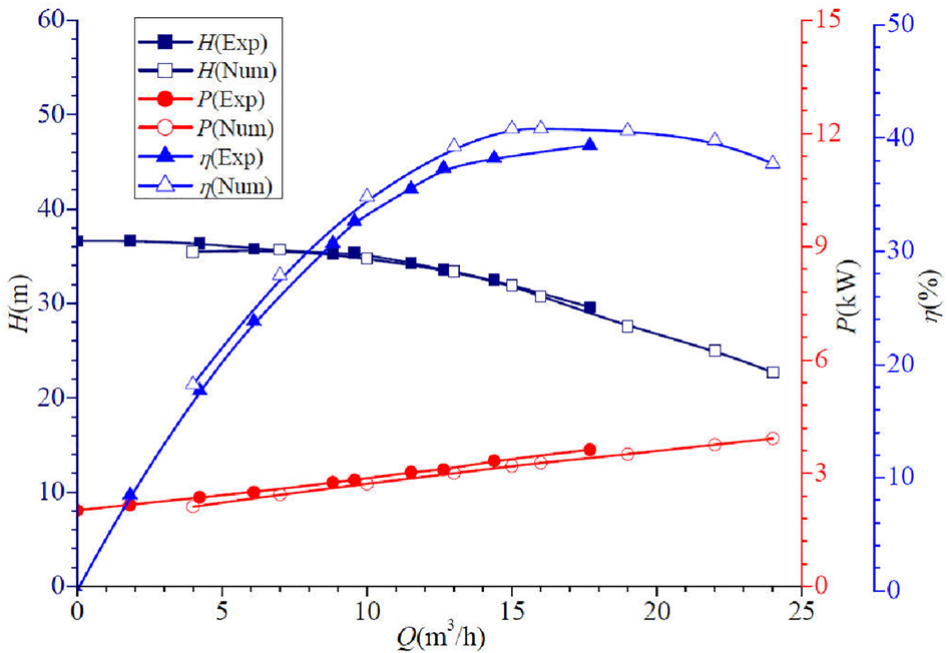
Comparison of hydraulic performance parameters of self-priming pumps.

The comparison between the hydraulic performance parameters of the self-priming pump under all flow conditions based on the above numerical calculation method and the experimental results is shown in Fig. 5. It can be seen that the numerical calculation results agree well with the experimental results. As the flow increases, both the test head and the calculated head gradually decrease. Under low and high flow conditions, the error between the test value and the calculated value is high, and the maximum difference in head is about 0.862 m, with an error of about 2.372%. The shaft power gradually increases with the flow rate, and exhibits a similar discrepancy to the head curve. Under low and high flow conditions, the error between the test value and the calculated value is high, and the maximum difference in shaft power is about 0.229 kW, accounting for about 9.778% of the test value. Furthermore, the efficiency curve calculated based on numerical simulation shows good agreement with experimental results. It shows an increase followed by a decrease, with a maximum efficiency value occurring near the design flow condition. The difference between the calculated and experimental results is within 8%. To sum up, the above numerical calculation methods are within acceptable limits, except under low flow conditions, confirming the high reliability of the above numerical calculation method. Based on this, subsequent numerical calculations will be carried out using this method.

## Orthogonal design scheme

### Determination of orthogonal design factors and levels

In combination with the structure of the self-priming pump and the relevant reference^6^, the key factors influencing the hydraulic performance of the self-priming pump are determined to be the impeller diameter (*D*_2_), the blade outlet width (*b*_2_), the blade wrap angle (*φ*) and the number of blades (*Z*), which are expressed as A, B, C and D respectively. The impeller diameter of the prototype pump is 166 mm, the blade outlet width is 8 mm, the blade wrap angle is 70° and the number of blades is 6. The levels of the influencing factors are determined according to the arithmetic progression rule, and the orthogonal design factors and level table are shown in Table 3. The orthogonal design scheme used in this paper is four factors and three levels, that is, the number of levels of the factors is 3, the number of factors is 4. Depending on the principle of orthogonality, it is determined that a total of 9 experiments need to be conducted. The corresponding orthogonal table is denoted as *L*_9_ (3^4^).

**Table 3:** Levels of factors in orthogonal experiments.

Level	Factor			
	(A) *D*_2_/(mm)	(B) *b*_2_/(mm)	(C) *φ*/(°)	(D) *Z*
1	166	9	80°	7
2	162	8	70°	6
3	158	7	60°	5

About factor A, the impeller diameter of the prototype self-priming pump is 166 mm. Theoretically, the prototype data should be used as a reference to increase and decrease the corresponding tolerance values as other levels. But considering the small height of the first section of the volute, increasing the diameter of the impeller will result in a smaller gap between the impeller and the volute tongue, which will lead to a more obvious effect of dynamic and static interaction between the rotor and the stator. This is not conducive to the stable operation of the pump. Therefore, reducing the tolerance of the prototype impeller size is taken as the new factor levels.

### Orthogonal design schemes and quality indicators

**Figure 6:**
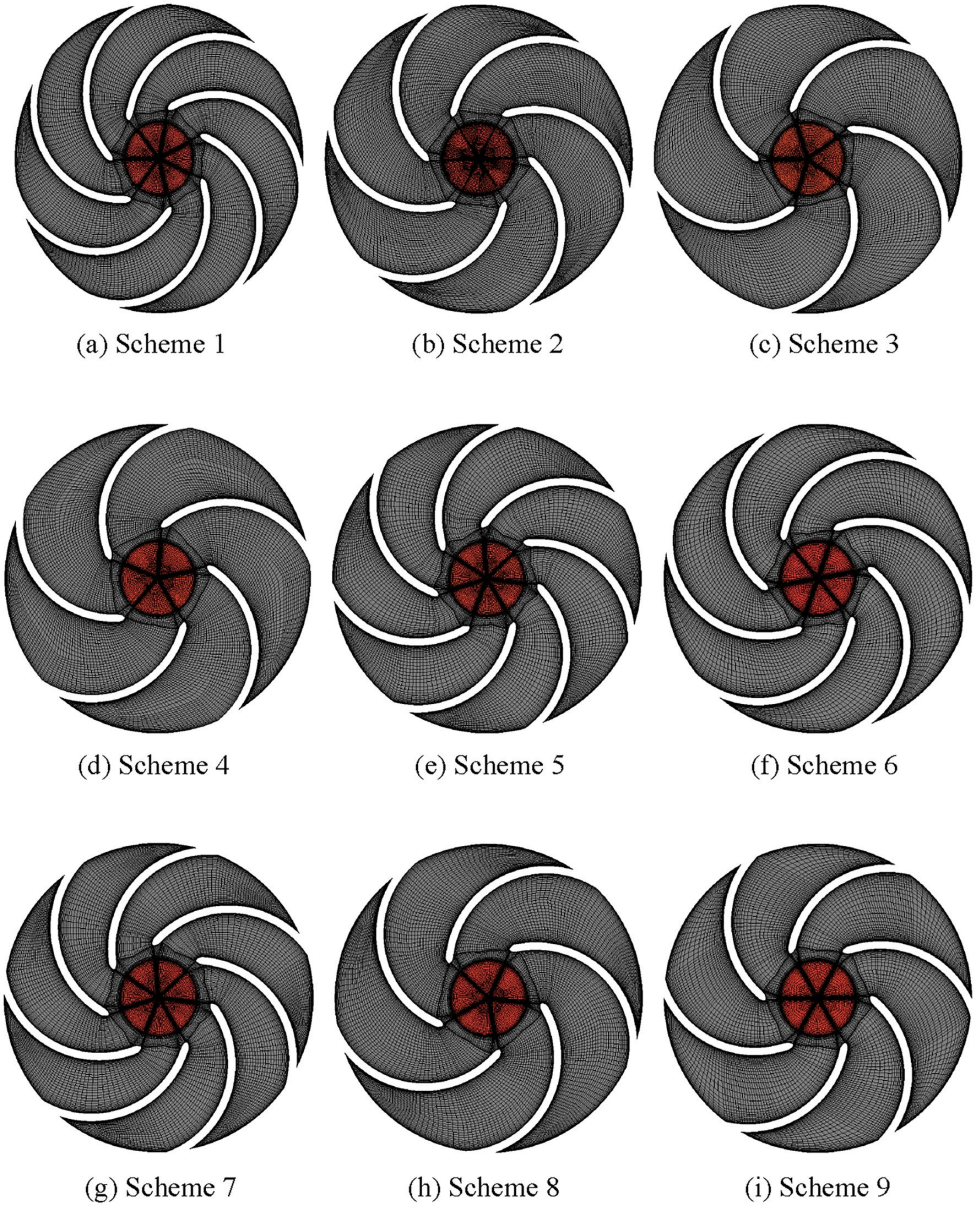
Impeller mesh of orthogonal test schemes.

The nine schemes based on the orthogonal Design of experiments are shown in Table 4, of which Scheme 2 is the parameter combination of the prototype pump. The quality indicators selected in this paper are the head and efficiency. Based on the parameter combinations in Table 4, the impellers in each of the nine test schemes are modeled and structurally meshed, and the meshes are shown in Fig. 6.

**Table 4:** Orthogonal test scheme.

Scheme ID	(A) *D*_2_/(mm)	(B) *b*_2_/(mm)	(C) *φ*/(°)	(D) *Z*	Test scheme
1	A_1_	B_1_	C_1_	D_1_	A_1_B_1_C_1_D_1_
2	A_1_	B_2_	C_2_	D_2_	A_1_B_2_C_2_D_2_
3	A_1_	B_3_	C_3_	D_3_	A_1_B_3_C_3_D_3_
4	A_2_	B_1_	C_2_	D_3_	A_2_B_1_C_2_D_3_
5	A_2_	B_2_	C_3_	D_1_	A_2_B_2_C_3_D_1_
6	A_2_	B_3_	C_1_	D_2_	A_2_B_3_C_1_D_2_
7	A_3_	B_1_	C_3_	D_2_	A_3_B_1_C_3_D_2_
8	A_3_	B_2_	C_1_	D_3_	A_3_B_2_C_1_D_3_
9	A_3_	B_3_	C_2_	D_1_	A_3_B_3_C_2_D_1_

## Analysis of results

### Hydraulic performance

**Figure 7:**
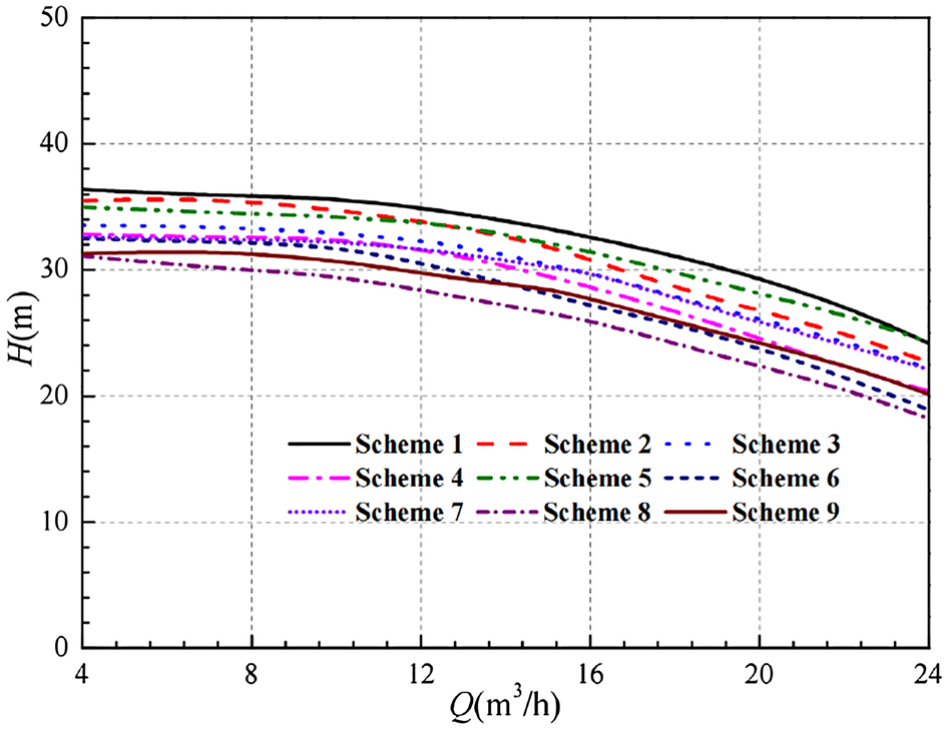
Comparison of orthogonal design head.

**Figure 8:**
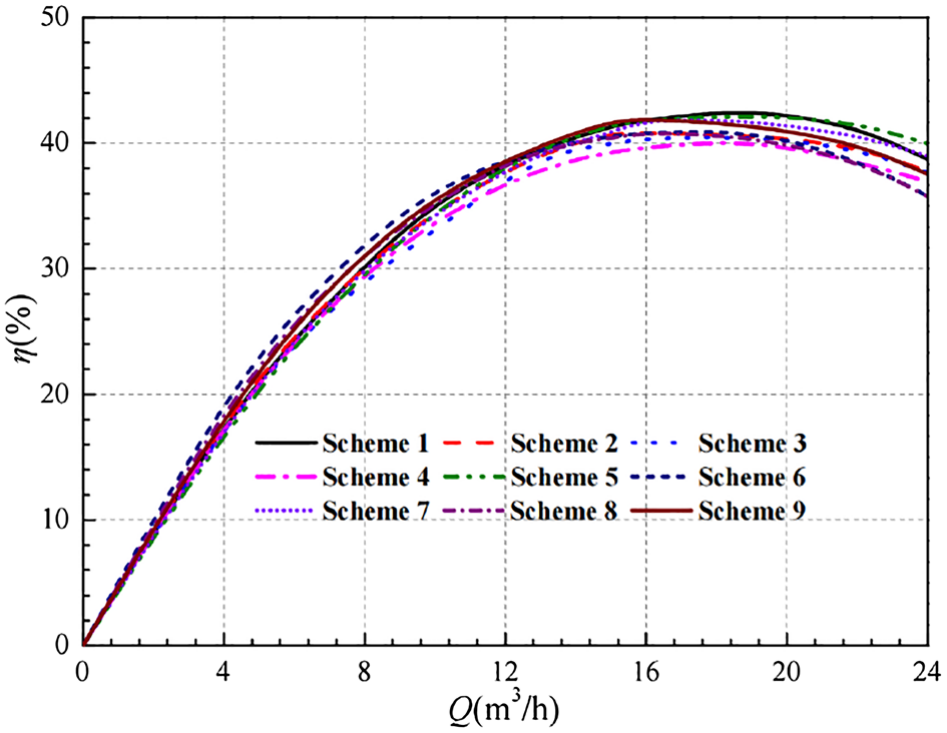
Comparison of orthogonal design efficiency.

The head and efficiency curves for various flow conditions under 9 schemes are shown in Figs. 7 and 8. It can be found that there are obvious differences in the head curves, but the trend of the curves remains the same, i.e., the head decreases gradually with the increase of the flow rate, and the decreasing trend becomes more obvious. The head result of Scheme 1 is the best, which is higher than other schemes in each flow condition, including the head curve of the prototype pump. Obviously, if the head alone is the only indicator, Scheme 1 is the optimal scheme.

Compared with the head curve, the impeller geometry parameters have a relatively small influence on the efficiency curve. At low flow conditions, the difference between the efficiency curves is relatively small. At high flow rate conditions, the difference in efficiency curves between the schemes becomes more obvious. Under the design flow conditions, the efficiency values of Scheme 1, Scheme 5 and Scheme 9 are very close, about 41.387%, 41.460% and 41.779%, respectively. And all of them are higher than the other six groups of schemes. However, under high flow conditions, it is not satisfactory that the efficiency of Scheme 9 has a significant decrease. In comparison with Scheme 1 and Scheme 5, Scheme 5 is higher. Therefore, if the head is the only indicator, Scheme 5 is the optimal scheme.

In summary, if both the head and efficiency are taken into account as indicators, the best scheme cannot be determined. The above analysis only compares the calculated data obtained from the designed 9 schemes and can only be used for preliminary judgment. The order of influence of each factor on the two quality indicators is now considered by range analysis.

### Range analysis

Combined with the orthogonal design principle, the hydraulic performance parameters of each scheme are analyzed for extreme differences under the design flow condition (15 m^3^/h). Table 5 shows the calculated results of the hydraulic performance of each scheme at its design flow rate.

**Table 5:** Calculation results of hydraulic performance.

Scheme ID	Test scheme	Head *H*/(m)	Efficiecy *η* /(%)
1	A_1_B_1_C_1_D_1_	33.304	41.387
2	A_1_B_2_C_2_D_2_	31.879	40.810
3	A_1_B_3_C_3_D_3_	30.362	39.946
4	A_2_B_1_C_2_D_3_	29.468	39.304
5	A_2_B_2_C_3_D_1_	32.062	41.460
6	A_2_B_3_C_1_D_2_	28.111	40.603
7	A_3_B_1_C_3_D_2_	30.178	40.757
8	A_3_B_2_C_1_D_3_	26.569	40.379
9	A_3_B_3_C_2_D_1_	28.515	41.779

In the range analysis, the sum of the test indicators (*K*), the mean of the test indicators (*k*) and the range of each quality indicator (*R*) should be calculated. Then the influence order of each factor on the quality indicators can be determined by using the range to assess their relative importance.

5 $$ k_{ij} = \overline{K}_{ij} = \frac{1}{n}\sum\limits_{i}^{n} {K_{ij} } $$

where *K*_*ij*_ is the sum of the quality indicators of the *j-*th factor corresponding to level *i*, $\overline{K}_{ij}$ is the average of *K*_*ij*_. And *i*, *j* are the level number and factor number respectively, *n* is the number of levels of the *j-*th factor.

6 $$ R_{j} = max\left\{ {\overline{K}_{ij} } \right\} - min\left\{ {\overline{K}_{ij} } \right\}\quad \left( {i = {1},{2}, \ldots ,{\text{n}}} \right) $$

where R_*j*_ is the range of the *j-*th factor. $max\left\{ {\overline{K}_{ij} } \right\}$, $min\left\{ {\overline{K}_{ij} } \right\}$ are the maximum and minimum values of the average of the quality indicator of the *j-*th factor, respectively.

Table 6 shows the range analysis of head and efficiency. It can be seen that if the head is the only indicator, the optimal combination of parameters is A_1_B_1_C_3_D_1_, which corresponds to the impeller diameter of 166 mm, blade outlet width of 9 mm, blade angle of 60° and the number of blades of 7. The influence order of each factor on the degree of head is: A > D > B > C, that is, the impeller diameter > the number of blades > the blade outlet width > the blade wrap angle. Similarly, if only efficiency is the only indicator, the optimal combination of parameters is A_3_B_2_C_1_D_1_. Which corresponds to the impeller diameter of 158 mm, the blade outlet width of 8 mm, blade angle of 80° and the number of blades of 7. The influence order of each factor on the efficiency is: A > D > B > C, that is, the number of blades > the impeller diameter > blade exit width > the blade wrap angle. In summary, it is found that the optimal combination of head and efficiency parameters do not all coincide, so further comprehensive analysis of the two indicators is required to select the best scheme that takes into account the optimal head and efficiency.

**Table 6:** Range analysis of head and efficiency.

Performance indicators		Factors			
		(A) *D*_2_	(B) *b*_2_	(C) *φ*	(C) *Z*
*H*/m	*K*_1*j*_	95.545	92.950	87.984	93.881
	*K*_2*j*_	89.641	90.510	89.862	90.168
	*K*_3*j*_	85.262	86.988	92.601	86.399
	*k*_1*j*_	31.848	30.983	29.328	31.294
	*k*_2*j*_	29.880	30.170	29.954	30.056
	*k*_3*j*_	28.421	28.996	30.867	28.800
	*R*_*j*_	3.427	1.987	1.539	2.494
*η*/%	*K*_1*j*_	122.143	121.448	122.368	124.626
	*K*_2*j*_	121.366	122.648	121.892	122.169
	*K*_3*j*_	122.914	122.327	122.164	119.628
	*k*_1*j*_	40.714	40.483	40.789	41.542
	*k*_2*j*_	40.455	40.883	40.631	40.723
	*k*_3*j*_	40.971	40.776	40.721	39.876
	*R*_*j*_	0.516	0.400	0.159	1.666

**Figure 9:**
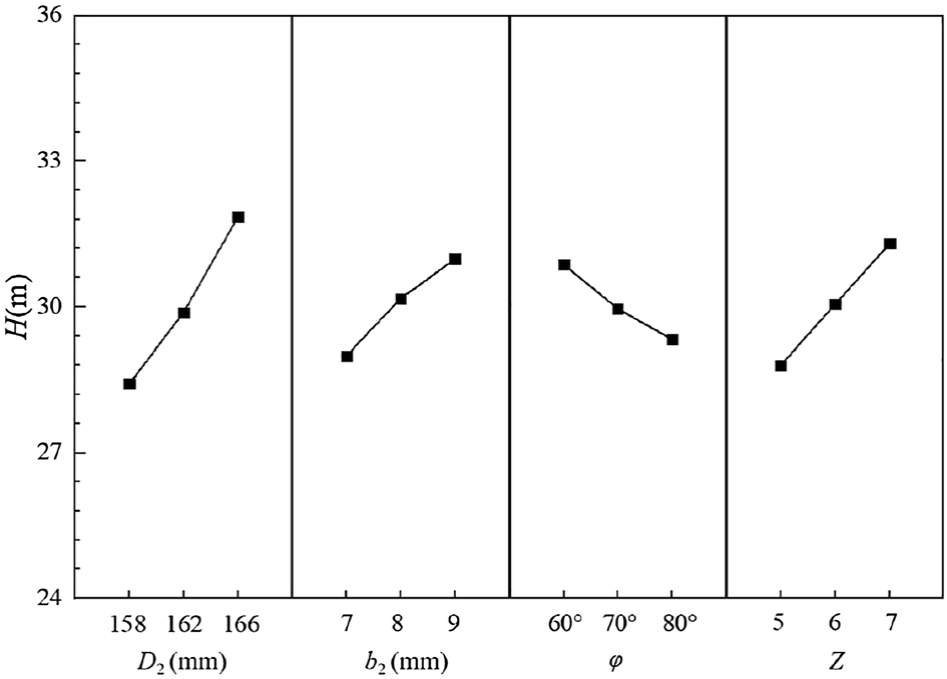
Relationship between head and factors.

**Figure 10:**
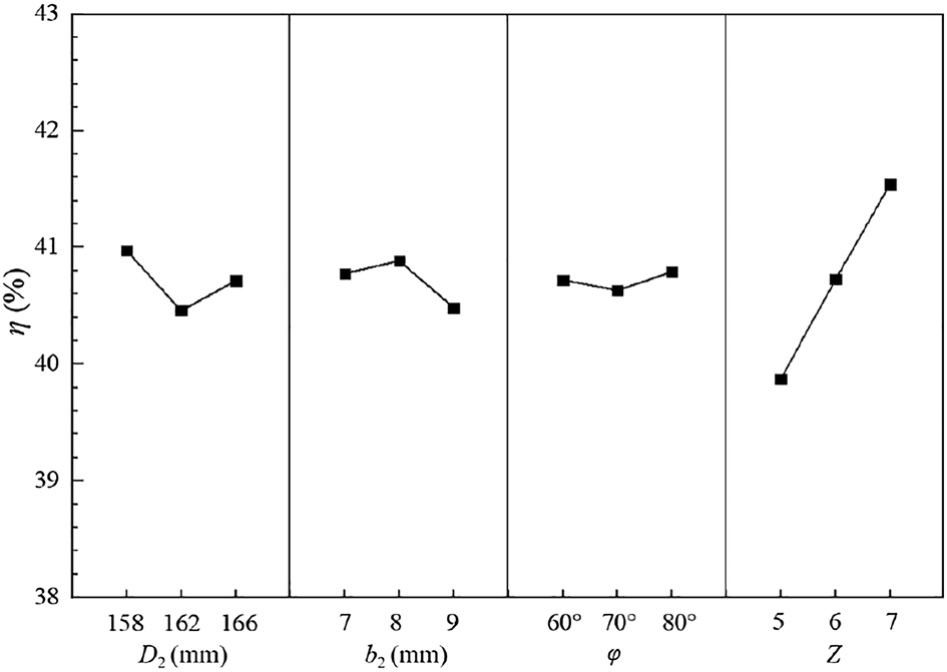
Relationship between efficiency and factors.

Figures 9 and 10 show the indicator-factor diagrams corresponding to head and efficiency respectively.

It can be found that the influence factor A, the impeller diameter, has a significant effect on the head. As the impeller diameter increases, the head gradually rises. Obviously, when the head indicator is considered alone, the larger impeller diameter is better. However, when considering the efficiency indicator, as the impeller diameter increases, the efficiency shows a trend of first decreasing and then increasing, and obviously the smaller impeller diameter is better. Combining the two quality indicators, when the impeller diameters are 158 mm and 166 mm, the corresponding efficiency indicator values are 40.971% and 40.714% respectively, with a small difference. But the values of the head indicator are 28.42 m and 31.85 m respectively, with a difference of 3.43 m. Under comprehensive analysis, the optimal value of the impeller diameter should be 166 mm, i.e. A_1_ level.

For the factor B, the blade outlet width, it can be seen that the change in factor B has a relatively small effect on head and efficiency, so it is a secondary factor for both quality indicators. When considering the head indicator alone, the larger blade outlet width provides a better head. When the blade outlet width is 8 mm and 9 mm, the corresponding efficiency indicator values are 40.883% and 40.483%, respectively. The difference is only 0.4%, and the relative proportion of the difference is 0.979%. While the head indicators are 30.17 m and 30.98 m, corresponding to a difference of 0.81 m, the relative proportion of 2.625%. Under comprehensive analysis, the optimal level of the blade outlet width should be 9 mm, i.e. B_1_ level.

For the factor C, the blade wrap angle, it can be seen that C is also a secondary factor for both quality indicators. When the head indicator is considered in isolation, the head decreases with increasing the blade wrap angle, with the smaller blade wrap angle providing a better head. But the efficiency indicator shows a trend of decreasing and then increasing with the increase of the blade wrap angle. Consistent with the analysis methods of the previous two factors, when the blade wrap angle is 60° and 80°, the corresponding head indicators are 30.87 m and 29.33 m respectively. The difference is 1.54 m, and the relative percentage of the difference is 4.986%. The corresponding efficiency indicators are 40.721% and 40.789%, with a difference of 0.068%, and the relative percentage is 0.167%. Under the comprehensive analysis, the optimal value of the blade wrap angle should be 60°, i.e. C_3_ level.

For the factor D, the number of blades, it can be seen that the factor D is more important for the two quality indicators. Both quality indicators increase gradually with the number of blades, so the optimal number of blades should be 7, i.e. D_1_ level.

In summary, the optimal combination of impeller parameters should be A_1_B_1_C_3_D_1_, corresponding to the following parameters: the impeller diameter of 166 mm, the blade outlet width of 9 mm, the blade wrap angle of 60° and the number of blades of 7.

However, this combination of parameters is not included in the initial orthogonal experimental design of nine schemes, so it is necessary to calculate the hydraulic performance of the self-priming pump for the A_1_B_1_C_3_D_1_ parameter combination.

### Result validation

**Figure 11:**
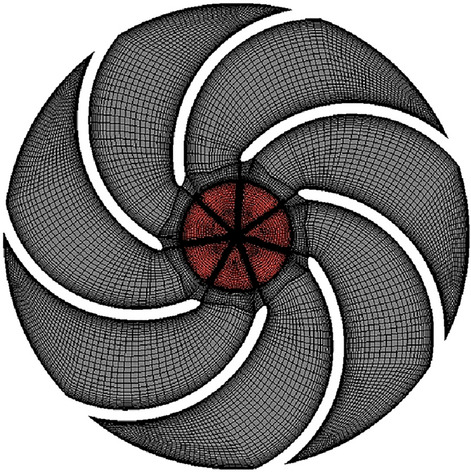
Optimal impeller mesh (Scheme 10).

According to the analysis of the factor indicators table in the orthogonal design, the optimal parameter combination A_1_B_1_C_3_D_1_ is derived. The impeller of the optimal parameter combination is modelled and structurally meshed, and its impeller mesh is shown in Fig. 11.

The numerical calculation data of the self-priming pump with this impeller is shown in Table 7.

**Table 7:** Hydraulic performance simulation results of the optimal scheme.

Flow *Q*/(m^3^/h)	Head *H*/(m)	Shaft Power *P*/(kW)	Efficiency *η*/(%)
4	36.369	2.335	16.929
7	36.480	2.595	26.740
10	36.270	2.914	33.822
13	35.030	3.224	38.382
15	34.155	3.420	40.706
16	33.434	3.525	41.237
19	30.933	3.773	42.329
22	28.205	4.046	41.674
24	26.196	4.257	40.132

**Figure 12:**
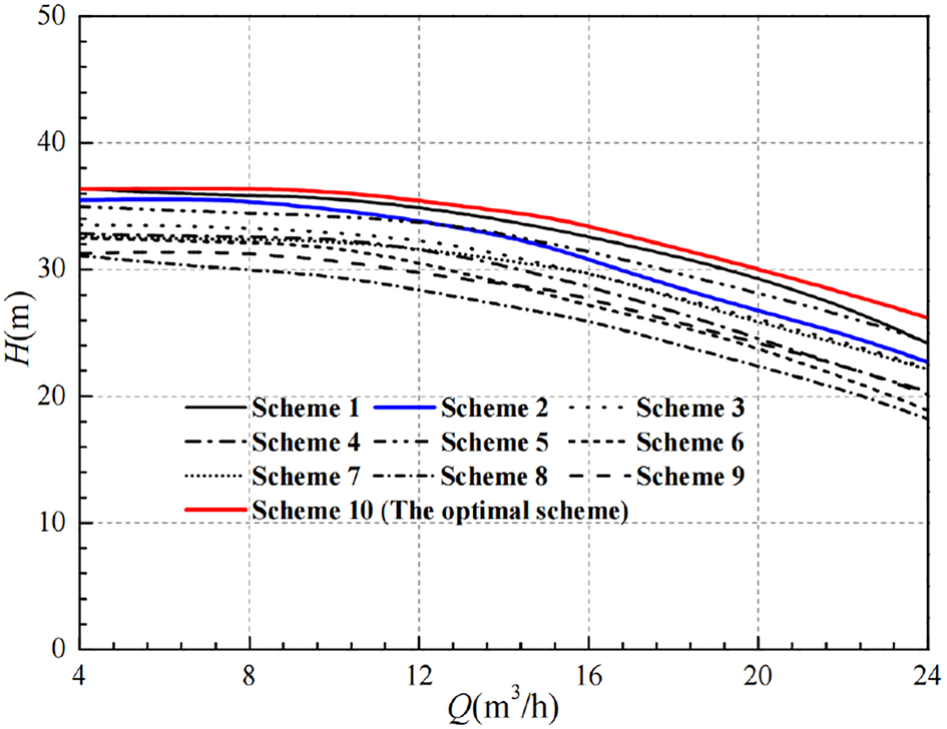
Comparison of head curves after optimisation.

**Figure 13:**
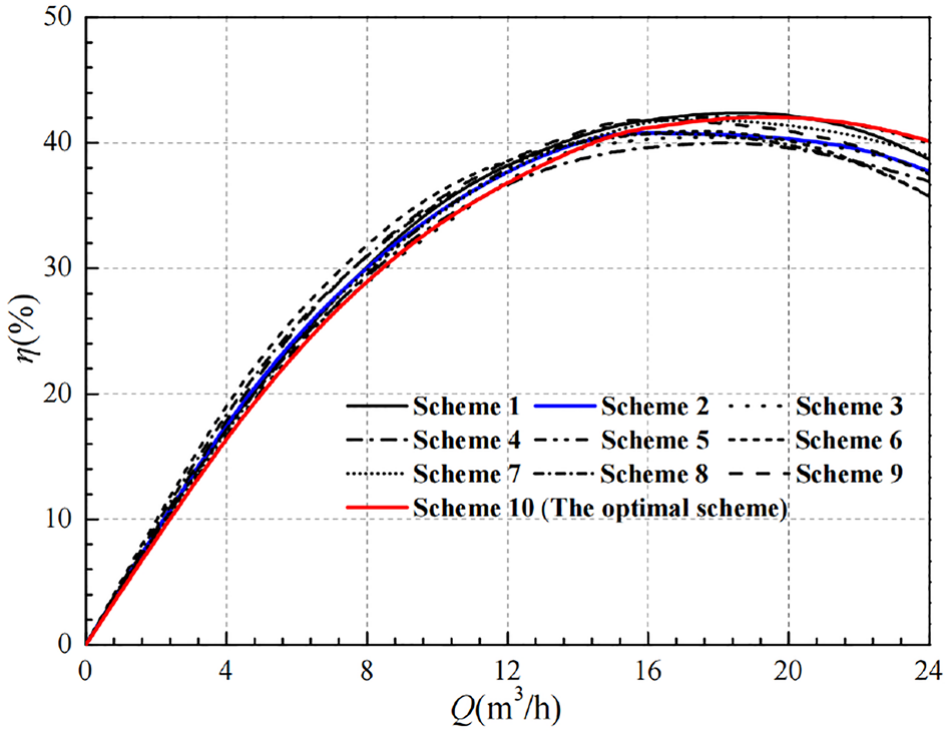
Comparison of efficiency curves after optimisation.

After the range analysis, the optimal parameters combination is determined to be Scheme 10, and its performance curves are compared in Figs. 12 and 13. It is easy to see that the numerical simulation results for Scheme 10 are all improved to some extent compared to the 9 schemes in the orthogonal experimental design.

Figure 12 shows that the head of the pump with the optimal parameter combination is higher than the other 9 schemes, including the prototype pump, under all flow rate operating conditions. At low flow conditions, the difference is small, while it is more obvious at high flow conditions. At the design flow rate *Q* = 15 m^3^/h, the head of Scheme 10 reaches 34.16 m, which is already higher than the design head of 32 m, indicating that the head indicator has met the design requirements.

Figure 13 shows that the efficiency of Scheme 10 is not optimal in the low flow range, but the difference in efficiency at the low flow conditions is very small overall. At the design flow condition, the efficiency of Scheme 10 is about 40.706%, which is slightly lower than that of the prototype pump at 40.810%. The best efficiency corresponds to Scheme 9 with the value of 41.779% and the difference of efficiency with Scheme 10 is only 1.073%, with a relative proportion of 2.568%. In addition, Scheme 10 has a clear advantage in terms of efficiency in the high flow range, and it also has a wide range of high efficiency zones.

In summary, Scheme 10 is more effective for raising the head. Under the small flow conditions, the reduction in efficiency is very small. But under the high flow conditions, the high efficiency zone is wider and has obvious advantages. Meanwhile, combined with the range analysis method, the optimal scheme can be determined as Scheme 10, with the specific parameters combination of A_1_B_1_C_3_D_1_, which corresponds to the impeller diameter of 166 mm, the blade outlet width of 9 mm, the blade wrap angle of 60°, and 7 blades. Through analysis of the simulation results, it is demonstrated that the optimization design of impellers based on orthogonal design principles and numerical simulation techniques has a certain guiding significance and can effectively reduce the cost and time of experimentation.

### Discussion

This paper is based on the orthogonal design principle and combines numerical calculations to optimise the design of the impeller, the core over-flow component of the self-priming pump. In the research process, this paper comprehensively considered the reflux hole, the front and rear pump chambers and the clearance of wear-ring on the hydraulic performance of the low specific speed self-priming pump. The design and calculation results have achieved the optimization purpose, but there are still some improvements that can be made.

1. In the orthogonal design process, the interactions between the influencing factors are ignored. This will need to be investigated in depth in future work.
2. In this paper, when determining the performance of self-priming pumps, the indicator of quality chosen is the hydraulic performance of the self-priming pump. As the research object is a self-priming pump, other parameters need to be studied in depth in future work, including self-priming height and self-priming time.

## Conclusion

1. This paper adopts the Reynolds-averaged Navier Stokes model and closes it with the Realizable k–ε turbulence model. The numerical calculation results have good agreement with the results of hydraulic performance experiments, verifying the accuracy of the numerical simulation method.
2. The order of factors for the degree of influence on the head is: *D*_2_ > *Z* > *b*_2_ > *φ*. The order of influence of each factor on the degree of efficiency is: *Z* > *D*_2_ > *b*_2_ > *φ*.
3. The optimal parameters combination is A_1_B_1_C_3_D_1_, namely *D*_2_ = 166 mm, *b*_2_ = 9 mm, *φ* = 60° and *Z* = 7. Under the design flow conditions, the corresponding head is 34.16 m, the shaft power is 3.42 kW and the efficiency is 40.706%. Compared to the prototype pump, the head is increased by 2.28 m, and the improvement in head is more significant under high flow conditions.
4. In the final optimized case, the difference in efficiency is very small at low flow conditions compared with 9 cases, but there is a significant enhancement in efficiency at high flow conditions, and with a wide high efficiency zone.

## Data Availability

The data used to support the findings of this study are available from the corresponding author upon request.
